# Sprouty2 correlates with favorable prognosis of gastric adenocarcinoma via suppressing FGFR2-induced ERK phosphorylation and cancer progression

**DOI:** 10.18632/oncotarget.13982

**Published:** 2016-12-16

**Authors:** Yunfei Xu, Xiaoqing Yang, Zhen Li, Shuo Li, Sen Guo, Sayed Ismail, Hongda Liu, Zhihong Huang, Zongli Zhang, Yuxin Chen, Qing Sun

**Affiliations:** ^1^ Department of General Surgery, Qilu Hospital Affiliated to Shandong University, Jinan, Shandong, China; ^2^ Department of Pathology, Qianfoshan Hospital Affiliated to Shandong University, Jinan, Shandong, China; ^3^ Department of Neurology, Yidu Central Hospital of Weifang City, Weifang, Shandong, China; ^4^ Department of Neurosurgery, Yidu Central Hospital of Weifang City, Weifang, Shandong, China; ^5^ 302 Hospital of People's Liberation Army, Beijing, China

**Keywords:** gastric adenocarcinoma, FGFR2, SPRY2, proliferation, invasion

## Abstract

Fibroblast growth factor receptor 2 (FGFR2) has been identified as a predictive biomarker for unfavorable prognosis of gastric adenocarcinoma. As a well-defined antagonist in FGFR2-induced RAS/ERK activation, ectopic expression of sprouty (SPRY) family was reported in several kinds of cancers except gastric cancer. To explore the clinical significance of SPRY family and its correlation with FGFR2, we detected the expression of FGFR2 and SPRY family in 104 cases of gastric adenocarcinoma and subsequently analyzed their correlations with clinicopathological factors and overall survival rates by univariate and multivariate analysis. As the result, we demonstrated that both FGFR2 high-expression and SPRY2 low-expression indicated poorer prognosis of gastric adenocarcinoma. SPRY2 low-expression was significantly associated with FGFR2 high-expression, positive lymphatic invasion and metastasis. We further proved that SPRY2 could suppress FGFR2-induced ERK phosphorylation, cell proliferation and invasion with experiments *in vitro* and *in vivo*. In conclusion, we demonstrated that SPRY2 low-expression is a biomarker for unfavorable prognosis in gastric adenocarcinoma. SPRY2 can antagonize FGFR2-induced proliferation and invasion via suppressing ERK phosphorylation in gastric cancer cells, indicating SPRY2 as a potential therapeutic target for gastric adenocarcinoma treatment.

## INTRODUCTION

Gastric cancer is one of the most common malignancies worldwide and the second most common cause of cancer-related deaths, accounting for nearly 1 in 10 of all cancer deaths [1]. Although many anticancer approaches such as trastuzumab have been applied, the prognosis of gastric cancer was still unsatisfactory. The 5-year overall survival rate ranges from 20% to 30% [2]. The important reasons resulting in the disappointing prognosis of gastric cancer include its silent clinical symptoms and high recurrence, so the identification of new predictive or prognostic biomarkers is urgently needed for early diagnosis and recurrence reduction.

In human beings, fibroblast growth factor receptor (FGFR) family consists of five members (FGFR1-5). They could regulate many cellular processes including proliferation, differentiation and mitosis by interacting with FGFs. A total of 18 FGFs exist in human and could bind to different FGFRs with different binding affinity. FGFRs can transduce signals after binding with FGFs and activate downstream molecules as kinases [3]. This diversity and flexibility of stimulation supplies a complicated FGF-FGFR network [4, 5]. FGFR2 is the only well-defined prognostic biomarker in gastric cancer among FGFR family. Massive previous clinical and experimental evidences demonstrated that FGFR2 is an unfavorable prognostic biomarker and antibodies targeting FGFR2 can suppress gastric cancer progression *in vivo* and *in vitro* [6–10].

The Sprouty (SPRY) family are key feedback inhibitors of FGFR-induced Ras/MAPK pathway [11]. SPRY family comprise of four isoforms (SPRY 1-4) with common conserved C-terminal Cysteine-rich domain and N-terminal tyrosine-containing sequence [12]. Ectopic expression or dysfunction of SPRY was proved to result in pathological conditions such as oncogenesis or cancer progression. SPRY is reported as a tumor suppressor in a variety of malignancies including breast cancer, liver cancer and prostate cancer, etc. [13–19]. However, the role of SPRY in several kinds of cancers is still controversial [20]. The role of SPRY in gastric cancer has not been explored till now, inspiring us to investigate the clinical significance of SPRY family in gastric adenocarcinoma.

Overall, although FGFR2 has attracted tremendous attention as a potential therapeutic candidate in gastric cancer, the function of SPRY, the key molecule inhibiting FGFR2 signaling, has not been well elucidated. This drives us to explore the expression and clinical significance of SPRY in gastric cancer. In our study, we investigated the expression of FGFR2 and SPRY family in 104 gastric adenocarcinoma cases and evaluated their clinical significance, including correlations with clinicopathologic factors and prognostic value. Additionally, we further explored the role of SPRY2 on progression of gastric cancer with experiments *in vitro* and *in vivo*.

## RESULTS

### Expression of FGFR2 and SPRY family in paraffin-bedded tissues

**Figure 1:**
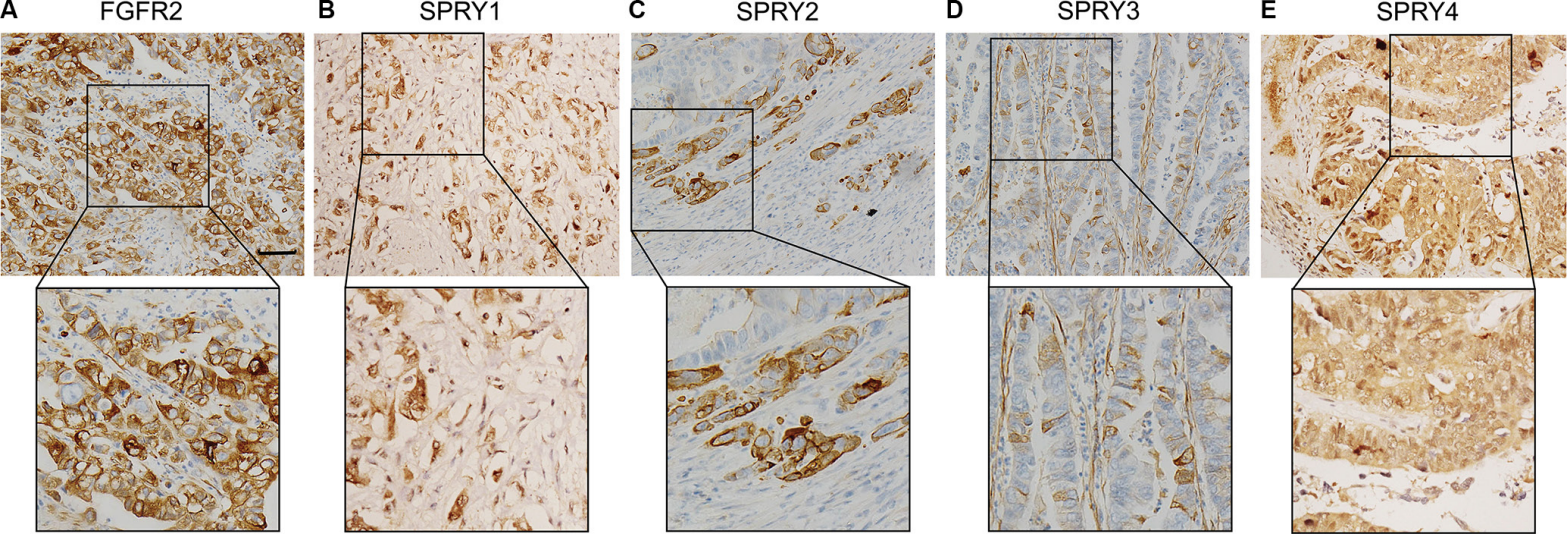
Representative figure of immunohistochemistry staining of high-expression of FGFR2 and SPRY family Expression of FGFR2 and SPRY family was detected by immunohistochemistry in formalin-fixed and paraffin-bedded gastric adenocarcinoma tissues. The cohort was divided into low-expression and high-expression according to the immunohistochemistry score. Representative immunohistochemistry image of the high-expression of (**A**) FGFR2, (**B**) SPRY1, (**C**) SPRY2, (**D**) SPRY3 and (**E**) SPRY4 were displayed. Scale bar: 50 μm.

Previous experiments demonstrated that SPRY1, SPRY2 and SPRY4 are expressed in various embryonic tissues, whereas SPRY3 is only detected in adult brain and mice testis [22, 23]. In our study, the expression of SPRY family and FGFR2 was detected with IHC in gastric adenocarcinoma tissues to examine their different expressive abundance. In our experiment, FGFR2, SPRY2 and SPRY3 were observed in both cytoplasm and membrane, while SPRY1 and SPRY4 mainly existed in cytoplasm (Figure 1). The cohort was divided into low-expression group and high-expression group as described in Materials and Methods. The proportion of high-expression rates of FGFR2, SPRY1, SPRY2, SPRY3 and SPRY4 were 36.54% (38/104), 15.38% (16/104), 32.69% (34/104), 4.81% (5/104) and 20.19 (21/104), respectively (Table 1).

**Table 1:** Characters of patients

Characters	Number	Percentage
Gender		
Male	66	63.46%
Female	38	36.54%
Age		
< 60	22	21.15%
≥ 60	82	78.85%
Tumor diameter (cm)		
≤ 5	31	29.81%
> 5	73	70.19%
Differentiation		
Well+Moderate	59	56.73%
Poor	45	43.27%
Tumor invasion		
T1 + T2	35	33.65%
T3 + T4	69	66.35%
Lymph node metastasis		
No (N0)	43	41.35%
Yes (N1/2/3)	61	58.65%
Distant metastasis		
M0	97	93.27%
M1	7	6.73%
TNM stage		
I	12	11.54%
II	31	29.81%
III	52	44.23%
IV	9	14.23%
Lauren subtype		
Intestinal	58	55.77%
Diffuse	34	32.69%
Mixed	12	11.54%
Peritoneal dissemination		
Yes	2	1.92%
No	102	98.08%
FGFR2		
low	66	63.46%
high	38	36.54%
SPRY1		
low	88	84.62%
high	16	15.38%
SPRY2		
low	70	67.30%
high	34	32.69%
SPRY3		
low	99	95.19%
high	5	4.81%
SPRY4		
low	83	79.81%
high	21	20.19%

Abbreviations: FGFR2 = fibroblast growth factor receptor 2, SPRY = sprouty.

### Correlation between FGFR2/SPRY2 and clinicopathologic factors

The correlation between FGFR2/SPRY2 and clinicopathologic factors was analyzed by Chi-square to screen the FGFR2/SPRY2-relevant factors (Table 2). FGFR2 high-expression was demonstrated to be significantly associated with positive lymphatic invasion (*P* = 0.007), Lauren subtype (*P* = 0.031) and lower SPRY2 expression (*P* = 0.029). Moreover, SPRY2 low-expression could indicate advanced T stage and positive lymphatic invasion (*P* = 0.002 and 0.001, respectively), suggesting the suppressive role of SPRY2 in gastric adenocarcinoma growth and invasion. Prognostic value of FGFR2 and SPRY family

**Table 2:** Correlations between clinicopathologic factors and FGFR2/SPRY2

Characters	FGFR2		P*	SPRY2		P*
	Low	High		Low	High	
Gender						
Male	41	25	0.833	44	22	1.00
Female	25	13		26	12	
Age						
< 60	13	9	0.628	17	5	0.314
≥ 60	53	29		53	29	
Tumor diameter (cm)						
≤ 5	19	12	0.825	20	11	0.820
> 5	47	26		50	12	
Differentiation						
Well + Moderate	38	21	0.840	41	18	0.674
Poor	28	17		29	16	
Tumor invasion						
T1 + T2	25	10	0.284	16	19	0.002
T3 + T4	41	28		54	15	
Lymph node metastasis						
No (N0)	34	9	0.007	21	22	0.001
Yes (N1/2/3)	32	29		49	12	
Distant metastasis						
M0	63	34	0.256	63	34	0.093
M1	3	4		7	0	
TNM stage						
I	8	4	0.701	7	5	0.217
II	17	14		17	14	
III	32	14		39	13	
IV	6	3		7	2	
Lauren subtype						
Intestinal	43	15	0.031	35	23	0.096
Diffuse	16	18		24	10	
Mixed	7	5		11	1	
Peritoneal dissemination						
Yes	64	38	0.532^#^	68	34	1.00^#^
No	2	0		2	0	
SPRY1						
low	54	34	0.401	61	27	0.386
high	12	4		9	7	
SPRY2						
low	39	31	0.029			
high	27	7				
SPRY3						
low	63	36	1.00^#^	66	33	1.00^#^
high	3	2		4	1	
SPRY4						
low	51	32	0.456	56	27	1.00
high	15	6		14	7	
FGFR2						
low				39	27	0.029
high				31	7	

* means calculated by Chi-square test; ^#^means calculated by Fisher test.

Abbreviations: FGFR2 = fibroblast growth factor receptor 2, SPRY = sprouty.

**Figure 2:**
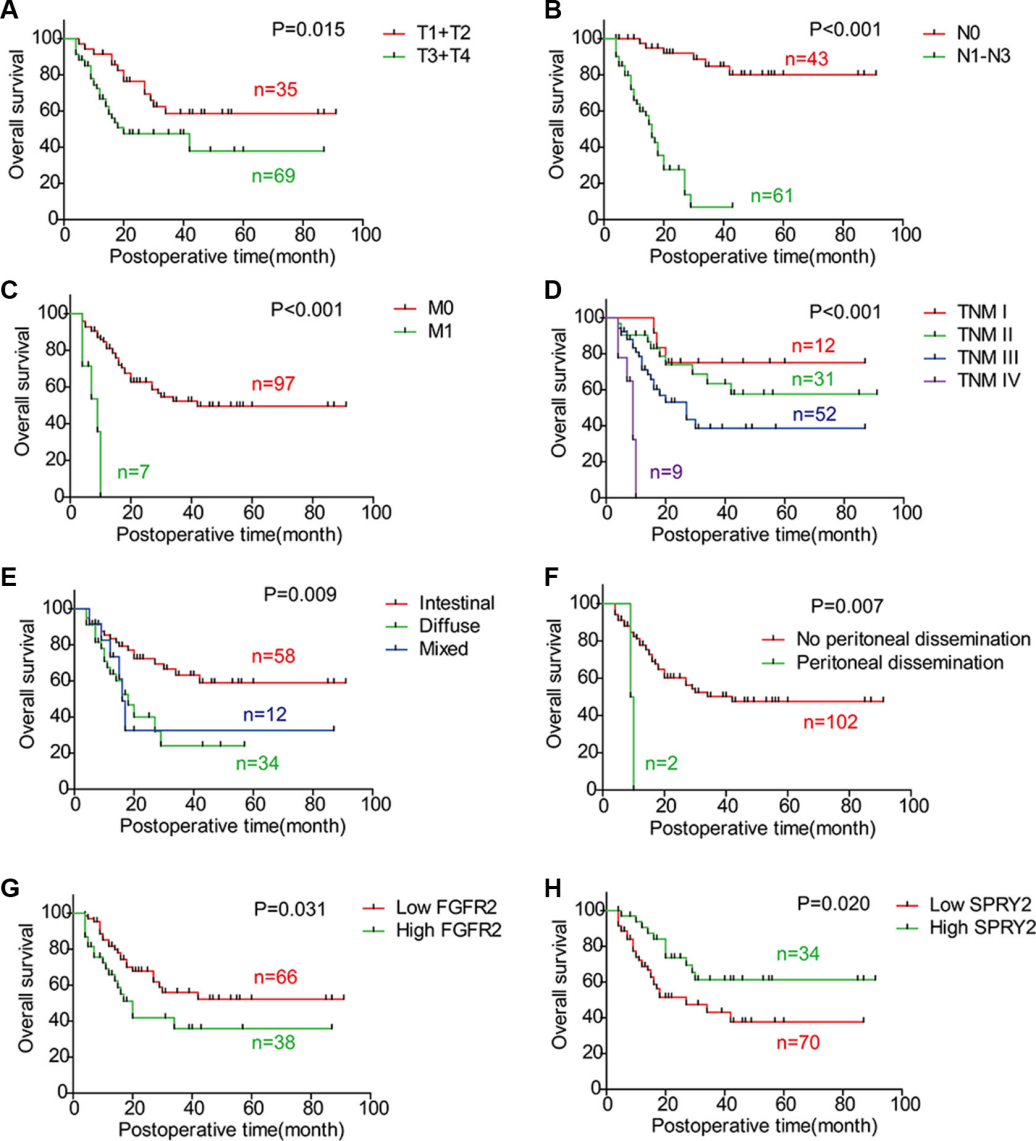
Correlations between overall survival rate and clinicopathologic factors The survival curves were graphed with Kaplan-Meier method and the statistical difference was analyzed with log-rank test. This figure displays the overall survival curves of (**A**) T1/T2 and T3/T4; (**B**) N0 and N1-3; (**C**) M0 and M1; (**D**) TNM stage; (**E**) Intestinal type, diffuse type and mixed type; (**F**) Negative and positive peritoneal dissemination; (**G**) FGFR2 low-expression and high-expression; (**H**) SPRY2 low-expression and high-expression.

To reveal the the clinical significance of FGFR2 and SPRY family, the overall survival rates of FGFR2 and SPRY family were investigated with univariate analysis (Table 3). With Kaplan-Meier method, advanced T stage, N stage, M stage and TNM stage were demonstrated to indicate unfavorable prognosis in our cohort (*P* = 0.015, *P <* 0.001, *P <* 0.001 and *P <* 0.001, respectively) (Figure 2A–2D). Diffuse and mixed Lauren subtype had poorer prognosis than intestinal subtype (*P* = 0.009) (Figure 2E), and patients with peritoneal dissemination had significantly lower survival rate than those without peritoneal dissemination (*P* = 0.007) (Figure 2F). Moreover, FGFR2 high-expression (*P* = 0.031, 5-year survival rate: 52.2% vs. 35.9%) (Figure 2G) and SPRY2 low-expression (*P* = 0.020, 5-year survival rate: 43.2% vs. 61.3%) (Figure 2H) were also demonstrated to be biomarkers for poorer prognosis in gastric adenocarcinoma.

**Table 3:** Prognostic value of clinicopatholgic factors and candidate biomarkers

Characters	Univariate analyisis		Multivariate analyisis		
	5-year survival rate	P*	HR	95%CI	*P*^#^
Gender					
Male	37.4	0.065			
Female	58.6				
Age					
< 60	49.6	0.666			
≥ 60	45.3				
Tumor diameter (cm)					
≤ 5	41.8	0.862			
> 5	49.6				
Differentiation					
Well + Moderate	57.7	0.124			
Poor	34.1				
Tumor invasion					
T1 + T2	58.6	0.015	1		
T3 + T4	37.9		1.97	0.92–4.19	0.079
Lymph node invasion					
No (N0)	80	*P <* 0.001	1		
Yes (N1/2/3)	6.9		15.6	5.42–45.3	*P <* 0.001
Distant metastasis					
M0	49.6	*P <* 0.001	1		
M1	0		4.15	1.55–11.1	0.005
TNM stage					
I	75	*P <* 0.001			
II	57.6				
III	38.6				
IV	0				
Lauren subtype					
Intestinal	59	0.009			
Diffuse	24				
Mixed	32.6				
Peritoneal dissemination					
Yes	0	0.007			
No	47.6				
FGFR2					
low	52.2	0.031	1		
high	35.9		1.22	0.65–2.26	0.537
SPRY1					
low	46.8	0.577			
high	46.8				
SPRY2					
low	43.2	0.020	1		
high	61.3		0.61	0.28–1.33	0.216
SPRY3					
low	47.1	0.737			
high	0				
SPRY4					
low	48.2	0.947			
high	39.5				

* means calculated by log-rank test, ^#^means calculated by Cox-regression hazard model,

Abbreviations: FGFR2 = fibroblast growth factor receptor 2, SPRY = sprouty, HR = hazard ratio, CI = confidence internal.

Multivariate analysis was further performed to identify independent prognostic biomarkers. Prognostic factors validated in univariate analysis were enrolled in Cox-regression model for multivariate analysis, including T stage, N stage, M stage, FGFR2 and SPRY2. TNM stage was excluded because of the interaction effect with T/N/M stage (Table 3). In multivariate analysis, N stage (*P <* 0.001, HR = 15.6, 95% CI = 5.42–45.3) and M stage (*P* = 0.005, HR = 4.15, 95% CI = 1.55–11.1) were identified as independent prognostic factors in gastric adenocarcinoma, while T stage tended to be an independent factor but this tendency is not statistically significant (*P* = 0.079). FGFR2 and SPRY2 were not confirmed as independent prognostic factors partially ascribed to the interaction effect with N stage and T stage, which was demonstrated in Chi-square test in Table 2 (*P* = 0.537 and 0.216, respectively).

### FGFR2, SPRY2 mRNA and microRNAs in tissues and cell lines

**Figure 3:**
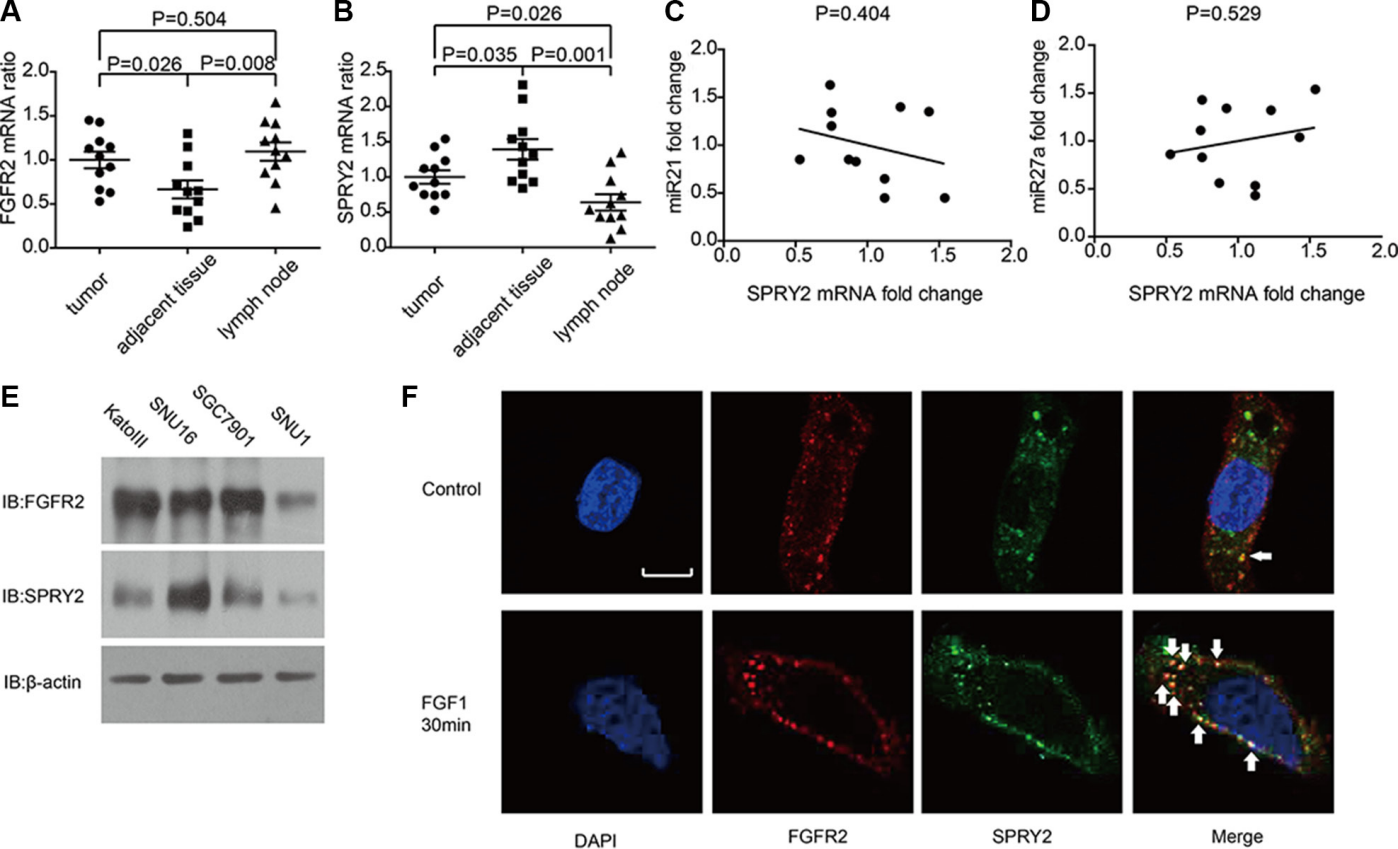
The mRNA level of FGFR2, SPRY2 and relative miRNAs in gastric adenocarcinoma tissues and cell lines (**A** and **B**)The mRNA levels of (A) FGFR2 and (B) SPRY2 in gastric adenocarcinoma tissues, adjacent tumor tissues and lymph nodes of 11 patients were detected by quantitative PCR with GAPDH as an internal control. The mRNA levels were standardized by ΔΔCt method. FGFR2 mRNA in adjacent tumor tissues was significantly lower than in tumor tissues (*P* = 0.026) and lymph nodes (*P* = 0.008), while SPRY2 mRNA in adjacent tumor tissues was significantly higher than in tumor tissues (*P* = 0.035) and invaded lymph node (*P* = 0.001). (**C** and **D**) The correlation between miR21, miR27a and SPRY2 mRNA. The quantification of miR21, miR27a and SPRY2 was realized by real-time PCR and standardized with the meanvalue set as 1. The correlation between miR21 and SPRY2 (C), between miR27a and SPRY2 (D) was analyzed by Pearson correlation coefficient. (**E**) FGFR2 and SPRY2 expression in gastric cell line KatoIII, SNU16, SGC7901 and SNU1 were detected by Western blotting. (**F**) The locations of FGFR2 and SPRY2 in SNU16 cells were indicated by immunofluorescence with/without FGF1 stimulation. Cell nucleus was stained by DAPI, while FGFR2 or SPRY2 was visualized after incubation with corresponding primary antibodies and secondary antibodies after starvation in serum-free medium overnight. Without FGF1 stimulation, FGFR2(red) was distributed around cell membrane and scattered in cytoplasm, and SPRY2 (green) expressed mostly in cytoplasm. After 10 ng/ml FGF1 stimulation for 30 minutes, FGFR2 and SPRY2 formed stimulation cluster in both membrane and cytoplasm. FGFR2 and SPRY2 had more co-localizations after FGF1 stimulation. Arrows pointed representative co-localizations. Scale bar: 5 μm.

The mRNA levels of FGFR2 and SPRY2 were detected by quantitative PCR in 11 samples of gastric adenocarcinoma, including tumor tissues, adjacent tumor tissues and invaded lymph nodes. The FGFR2 mRNA in adjacent tissues was remarkably lower than in tumor and lymphatic tissues, indicating FGFR2 could facilitate gastric cancer oncogenesis and invasion (Figure 3A). On the contrary, SPRY2 had significantly higher mRNA level in adjacent tissues compared with tumor or lymph node (Figure 3B), suggesting SPRY2 as a tumor suppressor in gastric adenocarcinoma. MiR-21 and miR-27a were proved to inhibit SPRY2 in previous studies [24, 25], so we also detected the levels of miR-21 and miR-27a in frozen tissues and analyzed their correlation with SPRY2. However, the level of miR-21 and miR-27a had no significant correlation with SPRY2 mRNA level in our study (Figure 3C and 3D).

The expression of SPRY2 in gastric adenocarcinoma cell lines including KatoIII, SNU16, SNU1 and SGC7901 were detected by Western blotting. KatoIII, SNU16 and SGC7901 had relatively higher FGFR2 expression while SNU16 had the highest SPRY2 expression (Figure 3E). Immunofluorescence was subsequently performed to confirm the expression and location of FGFR2/SPRY2 under FGF1 stimulation. In SNU16 cells, FGFR2 distributed around cell membrane and in the cytoplasm without FGF1 stimulation, with little co-localization with SPRY2. After 10 ng/ml FGF1 stimulation for 30 minutes, more co-localizations of FGFR2 and SPRY2 were observed, indicating SPRY2 may directly interact with FGFR2 complex after ligand activation (Figure 3F).

### SPRY2 could inhibit FGFR2-induced ERK phosphorylation

**Figure 4:**
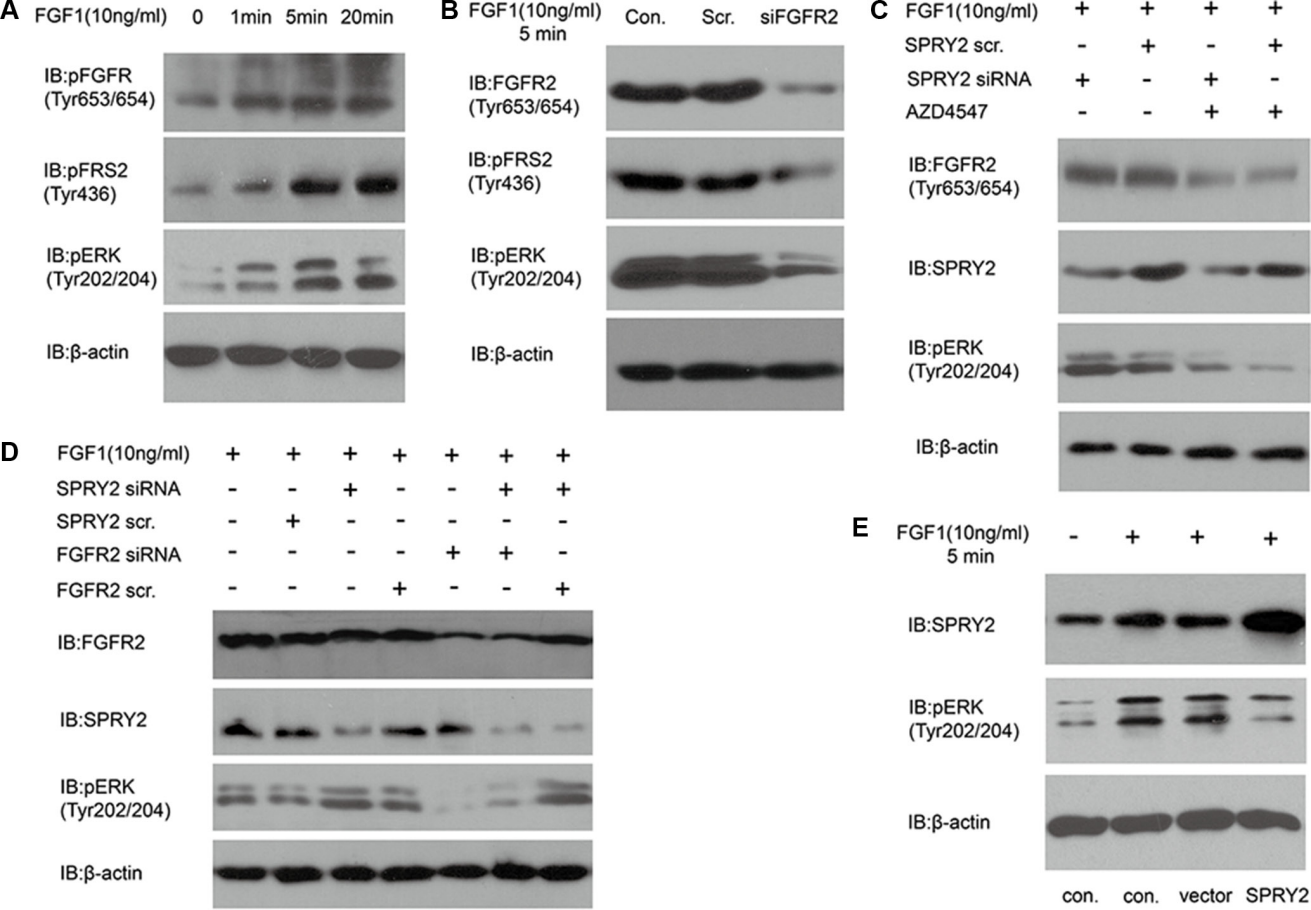
SPRY2 could antagonize FGFR2-induced ERK phosphorylation (**A**) The phosphorylation of FGFR (Tyr653/654), FRS2 (Tyr436) and ERK (Tyr202/204) increased along with FGF1 stimulation in KatoIII cells. Cells were starved in serum-free medium for 12 hours and then stimulated with 10 ng/ml FGF1 for 0–20 minutes before test. (**B**) Effect of FGFR2 knockdown on FRS and ERK phosphorylation. SNU16 cells were transfected with FGFR2 siRNA 48 hours before 10 ng/ml FGF1 stimulation for 5 minutes. Total FGFR2, phosphorylation of FRS2 (Tyr436) and ERK (Tyr202/204) was detected. Under FGF1 stimulation, phosphorylation of FRS2 and ERK decreased significantly after FGFR2 successful knockdown. (**C**) Effects of FGFR inhibitor AZD4547 on ERK phosphorylation with/without SPRY2 knockdown. SNU16 cells were incubated in AZD4547 (300 nM) for 2 hours before FGF1 (10 ng/ml) stimulation. SPRY2 siRNA knockdown could increase ERK (Tyr202/204) phosphorylation, and AZD4547 incubation suppressed ERK phosphorylation significantly by inhibiting phosphorylation of pFGFR (Tyr653/654). (**D**) ERK phosphorylation was attenuated by FGFR2 and enhanced by SPRY2 knockdown. Cells were transfected by SPRY2 siRNA and/or FGFR2 siRNA 48 hours before FGF1 stimulation. SPRY2 knockdown significantly elevated ERK phosphorylation and FGFR2 knockdown could reverse this effect. (**E**) SPRY2 overexpression could suppress ERK phosphorylation under FGF1 stimulation. SUN16 cells were transfected with FLAG-SPRY2 plasmid with empty pFLAG-CMV-2 vector as control. Cells were starved in serum-free medium overnight and then stimulated with 10 ng/ml FGF1. Con. means control group with only transfection agent. Vector represents the group transfected with empty pFLAG-CMV-2 vector. All results were confirmed with at least 3 independent experiments.

In Table 2, we proved that SPRY2 low-expression was significantly associated with FGFR2 high-expression and advanced T/N stage. Considering that SPRY2 was a well-acknowledged inhibitor of FGF-induced ERK phosphorylation, we evaluated the effect of SPRY2 on FGF-FGFR2 signaling with experiments *in vitro*. KatoIII cells were incubated in 10 ng/ml FGF1 for 0–20 minutes to detect FGF1 influence on FGFR downstream signaling. The phosphorylation of FGFR (Tyr653/654), FRS2 (Tyr436) and ERK (Tyr202/204) increased remarkably along with FGF1 incubation time (Figure 4A). After FGFR2 knockdown, SNU16 cells were incubated in 10 ng/ml FGF1 for 5 minutes and phopho-FRS2/ERK was detected. The phosphorylation of FRS2 and ERK decreased significantly in siFGFR2 group, indicating that FGFR2 was required in FGF1-induced FRS2 and ERK phosphorylation (Figure 4B). FGFR2 inhibitor AZD4547 was further used to detect FGFR2 effect on ERK phosphorylation (Figure 4C). SPRY2 knockdown significantly elevated ERK phosphorylation while AZD4547 decreased ERK phosphorylation. Further experiments with SPRY2/FGFR2 knockdown demonstrated that SPRY2 knockdown can evidently increase ERK phosphorylation but this effect was impaired when FGFR2 was knocked down concurrently, suggesting that SPRY2 could resist FGFR2-induced ERK phosphorylation (Figure 4D). Moreover, SPRY2 was overexpressed to confirm SPRY2 influence on ERC phosphorylation in circumstance of FGF1 stimulation (Figure 4E). Cells with FLAG-SPRY2 transfection had significantly higher SPRY expression but lower ERK phosphorylation (Figure 3F), which confirmed that SPRY2 inhibited FGF-induced ERK phosphorylation in gastric cancer cells.

### SPRY2 could suppress FGFR2-induced cell proliferation and invasion

**Figure 5:**
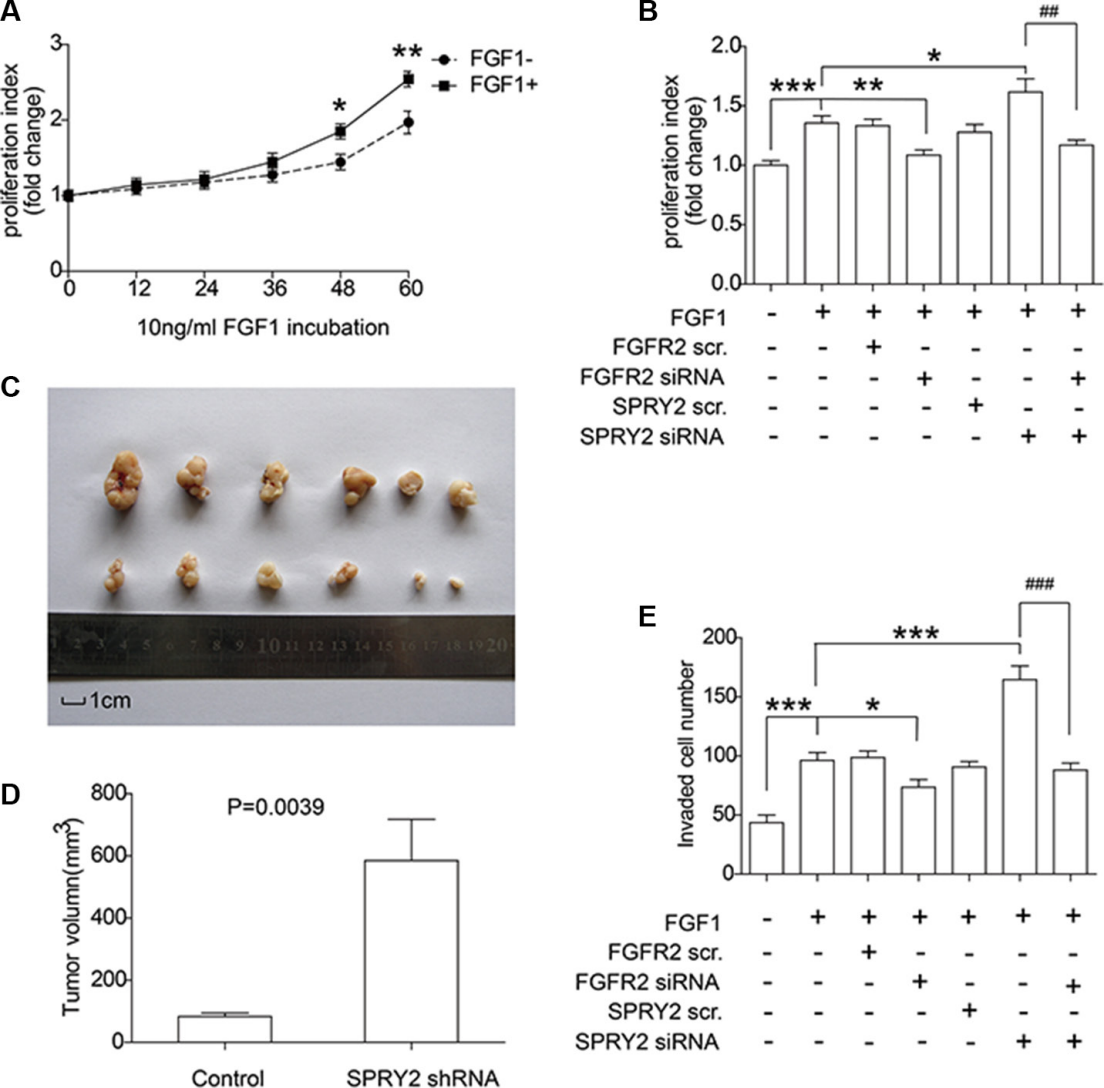
SPRY2 could suppress FGFR2-induced cell proliferation and invasion of gastric adenocarcinoma (**A**) SNU16 cells were incubated in FGF1 for 0 to 60 hours and the proliferation rate was quantified by MTT assay. Cells with FGF1 stimulation for 48 and 60 hours had remarkably higher proliferation rate than cells without FGF1 stimulation. *represents *P <* 0.05 and **represents *P <* 0.01 between FGF+ and FGF1- group at the same time. Data were from 3 independent experiments, analyzed by Student-*t* test. Error bar displays ± SEM. (**B**) Effect of FGFR2 and SPRY2 expression on gastric cancer cell proliferation. SNU16 cell proliferation was evaluated after 10 ng/ml FGF1 incubation for 48 hours and FGFR2 with/without SPRY2 knockdown. FGF1 could promote SNU16 proliferation via activating FGFR2, and SPRY2 could decrease FGFR2-promoted cell proliferation. *, ** and ***represents *P <* 0.05, *P <* 0.01 and *P <* 0.001 compared with the group only with FGF1 stimulation. ^##^means *P <* 0.01 between the linked columns. Data were from 3 independent experiments, analyzed by Student-*t* test. Error bar displays ± SEM. (**C**) Comparison of excised tumors volumn from xenograft model. SNU16 cells were first transfected with GV248-SPRY2 shRNA and selected with 1μg/ml puromycin before tumor injection. 10^6^ Stable SNU16/NC or SN U16/shRNA cells were injected subcutaneously into the left flank or right flank of BALB/C male nude mice. Upper row: tumors with SPRY2 knockdown; Lower row: tumors without SPRY2 knockdown. (**D**) Volume of tumor with/without SPRY2 knockdown 28 days after injection. The tumor volume (V) were calculated using the following formula: V (mm^3^) = V (mm^3^) = π × width (mm) × width (mm) × length (mm)/6. The *P value* was analyzed by Student's *t* test. (**E**) Cell invasion ability was promoted by SPRY2 knockdown and impaired by FGFR2 knockdown. SNU16 cell invasion was detected by transwell assay after 10 ng/ml FGF1 incubation for 48 hours and FGFR2 and/or SPRY2 knockdown. FGFR2 could promote cell invasion with FGF1 stimulation and SPRY2 could suppress this effect. * and ***represents *P <* 0.05 and *P <* 0.001 compared with the group with FGF1 stimulation and without other interferences. ^###^means *P <* 0.001 between the 6 and 7 columns. Data were from 3 independent experiments, analyzed by Student-*t* test. Error bar displays ± SEM.

Experiments *in vitro* and *in vivo* were performed to further explore FGFR2 and SPRY2 significance on gastric cancer progression. SNU16 cells were starved overnight and then incubated in 10 ng/ml FGF1 for 0–60 hours for MTT proliferation detection. FGF1 stimulation was proved to accelerate the proliferation of SNU16 cells after 48 hours stimulation (Figure 5A). Moreover, SPRY2 and/or FGFR2 knockdown was further performed to detect SPRY2/FGFR2 effect on cell proliferation. SNU16 cells were starvated in serum-free medium overnight and incubated in FGF1 for 48 hours before test. FGFR2 knockdown could significantly decrease cell proliferation while SPRY2 siRNA promoted proliferation significantly, indicating that SPRY2 could inhibit FGFR2-induced proliferation (Figure 5B). In addition, xenograft model was established to evaluate SPRY2 role in tumor growth. Stable SNU16 SPRY2-knockdown cells were realized by transfection with GV248-SPRY2 shRNA and selected by puromycin. Cells with/without SPRY2 knockdown were injected subcutaneously and harvested for 4 weeks. In our study, tumors with SPRY2 knockdown (upper row, Figure 5C) had remarkably larger sizes than these without SPRY2 knockdown (lower row, Figure 5C) (Figure 5D), confirming the suppressor role of SPRY2 in gastric tumor growth.

Invasive ability of SNU16 cells was detected by transwell assay with SPRY2 and/or FGFR2 knockdown (Figure 5E). FGF1 stimulation facilitated cell invasion while FGFR2 knockdown decreased cell invasive ability, demonstrating that FGFR2 was required in FGF-induced invasion. SPRY2 knockdown significantly enhanced cell invasion, but this enhancement was reversed when FGFR2 was concurrently knocked down, demonstrating the contrary effect of SPRY2 and FGFR2 on cell invasion. In summary, above observations suggested that FGFR2 could facilitate cell proliferation and invasion in gastric cancer, and SPRY2 functioned as an antagonism in this process.

## DISCUSSION

Since the first discovery of SPRY in Drosophila, it was considered as a negative modulator of Ras/MAPK pathway. The inhibitory effect of SPRY on Ras/MAPK pathway had selectivity on activation initiator, mostly the growth factors [26]. It was reported that SPRY2 and SPRY4 inhibit FGF-induced ERK signaling, but barely suppress EGF/PDBu-induced ERK phosphorylation in HEK293 cells [27]. This specific inhibition suggested that SPRY interacted with upstream proteins instead of catalyzing ERK directly, but it is still controversy now [28–30]. In our experiments, we observed more co-localizations of FGFR2 and SPRY2 after FGF1 stimulation. This supported that SPRY2 could interact with FGFR2 complexes such as FGFR2-FRS-Grb2-SOS directly.

Deregulation of SPRY was reported in many kinds of cancers [31]. SPRY is considered as a tumor suppressor, but its role in cancer is still controversy. Some sporadic studies reported that SPRY had oncogenic effects in some tumors. For example, SPRY was reported to have an atypical and dual role in colorectal cancer. In colorectal cancer, SPRY2 was proved to repress epithelial-mesenchymal transition and cell proliferation [20], while some other evidence proved that SPRY2 high-expression indicated poorer prognosis [32]. This paradox may be resulted from that SPRY was a negative regulator of FGF and other growth factors, but a positive enhancer of EGF signaling [33]. We proved that SPRY2 is an unfavourable biomarker for gastric cancer prognosis in our study. This may be a supplement to existing controversy of SPRY2 in cancer and help reveal more underlying function of SPRY2.

In summary, we detected a series of biomarkers in 104 cases of gastric adenocarcinomas, including SPRY family and an identified biomarker-FGFR2. As the result, we demonstrated that FGFR2 high-expression and SPRY2 low-expression were significantly associated with unfavorable prognosis of gastric adenocarcinoma. We also proved that SPRY2 could inhibit FGFR2-induced ERK phosphorylation and suppress FGFR2-elicited gastric cancer cell proliferation and invasion. We hope our findings could expand the understanding of SPRY2 in gastric adenocarcinoma, help find a new chemotherapy and improve the survival rates for patients with gastric cancer.

## MATERIALS AND METHODS

### Patients information

A total of 464 patients were diagnosed as gastric adenocarcinoma and radical gastrectomy from 2002 to 2013 in Qilu Hospital and Qianfoshan Hospital affiliated to Shandong University, and Yidu Central Hospital affiliated to Weifang Medical School, composing the primary cohort. From the primary cohort, the validation cohort including 104 cases was selected according to the criteria as follows: (i) available formalin-fixed tumor tissues, (ii) available clinical follow-up data and complete medical records, (iii) no history of previous anticancer therapy and other malignancies. Moreover, 11 pairs of tumor tissues and the corresponding adjacent tissues were collected and preserved in liquid nitrogen immediately after surgical resection for quantitative PCR(qPCR) detection. All the formalin-fixed and the fresh frozen tissues were obtained with prior consent of patients and approval of the Institutional Clinical Ethics Review Board. Pathologic tumor-node-metastasis (pTNM) staging was based on the 7th staging classification of AJCC/UICC (2009). The protocol of this study was managed according to the requirement of Reporting Recommendations for Tumor Marker Prognostic Studies (REMARK) [21].

### Cell culture and reagents

Detailed in Supplementary Materials.

### Immunohistochemistry

Detailed in Supplementary Materials.

### Quantitative PCR analysis of FGFR2 and SPRY2

Detailed in Supplementary Materials.

### Gene knockdown, amplification and transfection

Detailed in Supplementary Materials.

### Cell proliferation assay

Detailed in Supplementary Materials.

### Matrigel invasion assay

Detailed in Supplementary Materials.

### Western blotting

Detailed in Supplementary Materials.

### Immunofluorescence

Detailed in Supplementary Materials.

### MicroRNA detection

Detailed in Supplementary Materials.

### Xenograft model

Detailed in Supplementary Materials.

### Statistical analysis

All the data were analyzed by SPSS 17.0 software (IBM Corporation, USA). Software Graphpad Prism was also used to analyze data and graph figures. The correlation between biomarker expression and clinicopathologic features was assessed by χ^2^ test. The survival curve was evaluated in the Kaplan-Meier method, and the difference between high and low expression was calculated in a log-rank test. Cox proportional hazards regression model was used to identify the independent prognostic marker. In cell proliferation or invasion assay, student-*t* test was used to analyze the difference between different groups without special instruction. *P*-values < 0.05 was considered to be statistically significant.

## SUPPLEMENTARY MATERIALS


