# Supplementary material for: Sprouty2 correlates with favorable prognosis of gastric adenocarcinoma via suppressing FGFR2-induced ERK phosphorylation and cancer progression
Source: Oncotarget. 2016 Dec 16;8(3):4888–900. doi: 10.18632/oncotarget.13982 (PMC5354878; doi:10.18632/oncotarget.13982)
Supplement: Supplementary file 1 [file oncotarget-08-4888-s001.pdf]

Sprouty2 correlates with favorable prognosis of gastric adenocarcinoma via suppressing FGFR2-induced ERK phosphorylation and cancer progression

Supplementary Materials

MATERIALS AND METHODS

Cell culture and reagents

Gastric adenocarcinoma cell lines SNU1, SNU16, KatoIII, and SGC7901 were all purchased from American Type Culture Collection (Manassas, VA, USA) or Cell Bank of the Chinese Academy of Sciences (Shanghai, China). All cells were cultured in the RPMI-1640 medium supplemented with 10% fetal bovine serum (Gibco, USA) and 100 U/ml Penicillin and 100 μg/ml Streptomycin (HyClone, USA) in 5% CO_2 resuscitation.

FGF1 was purchased from PeproTech Company (Rocky Hill, NJ, USA), and all reagents without special instruction were purchased from Sigma-Aldrich Corporation. The following antibodies were used: FGFR2 (Cat. No. ab58201, Abcam, Cambridge, UK), phospho-FGFR (tyr653/654) (Cat. No. 3471, Cell Signaling Technology, Beverly, MA, USA), phospho-FRS2 (tyr436) (Cat. No. 3861, Cell Signaling Technology Beverly, MA, USA), Phospho-p44/42 MAPK (ERK1/2) (Thr202/Tyr204) (Cat. No. 4370, Cell Signaling Technology, Beverly, MA, USA), SPRY1(Cat. No. ab111523, Abcam, Cambridge, UK), SPRY2(Cat. No. ab60719, Abcam, Cambridge, UK), SPRY3(Cat. No. MBS2014353, MyBioSource, San Diego, CA, USA), SPRY4(Cat. No. ab7513, Abcam, Cambridge, UK).

Immunohistochemistry

The streptavidin peroxidase complex method was used for immunohistochemistry(IHC) as previously described [23]. Negative controls (absence of primary or secondary antibody) were applied for quality control. The immunohistochemical staining was evaluated independently by two senior pathologists unaware of clinical information, and cases without consensus were re-evaluated by the third pathologist. The immunohistochemical results were calculated by the score of percentage of positive-stained cells multiplied by staining intensity. The staining intensity was scored as negative (0), weak (1), moderate (2) and strong (3); and percentage of positive-stained tumor cells was defined as follows: 1, < 25% of positive cells; 2, 25%–50% of positive cells; 3, 50%–75% of positive cells; 4, 75%–100% of positive cells. The cohort was divided into low-expression group and high-expression group according to the cut-off, which was calculated by ROC curves and selected as the score of highest specificity plus sensitivity.

Quantitative PCR analysis of FGFR2 and SPRY2

Quantitative PCR was used to detect the mRNA levels of FGFR2 and SPRY2 in gastric adenocarcinoma tissues and adjacent tissues. Total mRNA was first extracted by Trizol agent (Invitrogen, Foster City, CA, USA) and RNeasy protect mini kit (Qiagen, Hilden, Germany). Complementary DNA (cDNA) synthesis and quantitative PCR was realized with SYBR Green Master Mix and StepOnePlus system (Applied Biosystem, Waltham, Massachusetts, USA) according to the manual. Relative expression was calculated by the ΔΔCt method. GAPDH (glyceraldehyde 3-phosphate dehydrogenase) was an internal control for the test to standardize the expression levels. The primers were designed as follows:

FGFR2, forward 5′-CAGTAGGACTGTAGACAG TGAA-3′, reverse 5′-CCGGTGAGGCGATCGCTCC ACA-3′; SPRY2, forward 5′-CCCCTCTGTCCAGAT CCATA-3′, reverse 5′-CCCAAATCTTCTTGCTCAG-3′; GAPDH, forward 5′-GAGTCAACGGATTTGGTCGT-3′, reverse 5′-GACAAGCTTCCCGTTCTCAG-3′.

Gene knockdown, amplification and transfection

Human SPRY2 open-reading-frame plasmid was purchased from Sino Biological Incorporation (Beijing, China), and subcloned into the pFLAG-CMV-2 vector by double-enzyme digestion. Knockdown of FGFR2 and SPRY2 were performed with small interfering RNA (siRNA) purchased from Dharmacon (Lafayette, CO, USA) and Santa Cruz Biotechnology (Santa Cruz, CA, USA), respectively. Transfection of siRNA or vector was accomplished by Lipofectamine 2000 (Invitrogen, Waltham, Massachusetts, USA) according to the guideline.

Cell proliferation assay

The proliferation of gastric adenocarcinoma cell line was evaluated by MTT assay as described before [23]. Briefly, cells were passaged into 96-well plates at density of 4,000/well and starved for at least 6 hours before stimulation. Human recombinant FGF1 at 100 ng/ml may be applied if necessary in experiment in 1%-serum-containing medium. At the end of stimulation, 10 μl MTT

at concentration of 5mg/ml was added and optical density (OD) at 490 nm was measured in a spectrophotometer (Molecular Devices Company, USA). The OD490 of corresponding control group was set as a baseline and OD490 of other groups was normalized by ratio to control group.

Matrigel invasion assay

Matrigel-precoated transwells (BD Biosciences, Franklin Lakes, NJ, USA) was used for transwell invasion assay as previously instructed [24]. Briefly, cells were passaged into the upper matrigel-coated chambers. Medium of upper chamber was changed into serum-free medium with 1%-serum-containing medium in lower chamber as chemotaxin. After incubation for 24 hours, cells were fixed and stained. At least 5 random visual fields under microscope were selected for cell counting.

Western blotting

The protein expression of gastric tumor cells was detected by Western blotting. Cells were first lysed in lysis buffer (1% NP-40 10 mM Tris-HCl, pH 7.4, 150 mM NaCl, 5 mM EDTA, 1 mM sodium vanadate, and 10 μg of leupeptin, 1 μg of aprotinin, 1 μg of pepstatin, 1 μg of antipain, and 30 μg of phenylmethylsulfonyl fluoride per ml) and centrifuged at 10000 × g for 30 minutes. The supernatant was collected and mixed with 2× sample buffer. After electrophoresis in SDS-polyacrylamide gel electrophoresis (SDS-PAGE), proteins were transferred to polyvinylidene difluoride (PVDF) membrane (Millipore, Billerica, MA, USA). 5% fat-free milk was used for antigen blocking, followed by primary antibody overnight, secondary antibody for 2 hours and enhanced chemiluminescence in sequential order.

Immunofluorescence

Immunofluorescence was performed to detect FGFR2 and SPRY2 expression and location in gastric cancer cells. SNU16 cells were passaged on coverslips in 24-well plates and starved in serum-free medium overnight before test. Cells were then fixed for 10 minutes in 4% paraformaldehyde at room temperature, and incubated in PBS with 0.5% Triton X for 2–5 minutes for membrane perforation. After antigen blockage in goat serum for 30 minutes, cells were incubated in primary antibody (1:50) at 4°C overnight and in secondary antibody at room temperature for 1 hour. Finally, cells were stained with 4′,6-diamidino-2-phenylindole (DAPI) (1:2000) for 5 minutes and mounted in Prolong Gold (Invitrogen). Final cell immunofluorescence was observed with confocal microscopy LSM780 (Zeiss Corporation, Germany).

MicroRNA detection

Stem-loop real-time RT-PCR was used for the detection and quantification of mature miRNA including miR-21 and miR-27a, with U6 as endogenous control. The sequences of the primers were as follows:

miR-21-RT: 5′-CTCAACTGGTGTCGTGGAGTC GGCAATTCAGTTGAGTCAACATC-3′. miR-27a-RT: 5′-GTCGTATCCAGTGCAGGGTCCGAGGTATTCGC ACTGGATACGACGCGGAA-3′; U6-RT:5′-CGCTTCA CGAATTTGCGTGTCAT-3′.

The cDNA generated from reverse-transcription PCR was used for detection of miR-27a, miR-21and U6. The sequence of qPCR primers were as follows:
miR-27a-F: 5′-TTCACAGTGGCTAAG-3′;
miR-27a-R: 5′- GTGCAGGGTCCGAGGT-3′;
miR-21-F: 5′-ACACTCCAGCTGGGTAGCTTATCAGAC TGA-3′.
miR-21-R: 5′-GTGTCGTGGAGTCGGCAATTC-3′.
U6-F: 5′-GCTTCGGCAGCACATATACTAAAAT-3′;
U6-R: 5′-CGCTTCACGAATTTGCGTGTCAT-3′;

Real time PCR was performed with SYBR Green (Applied Biosystem, Waltham, Massachusetts, USA) method according to the manual.

Xenograft model

All mice were purchased from Beijing Laboratory Animal Research Center (Beijing, China), and all animal procedures were approved by the Animal Management Committee of Shandong University. SNU16 cells were first transfected with GV248-SPRY2 shRNA and selected with 1 ug/ml puromycin before tumor injection. After successful knockdown and puromycin selection, 10^6 cells with/without SPRY2-knockdown were injected subcutaneously into the left flank or right flank of BALB/C male nude mice weighing 16–18 g at 6–8 weeks of age. Tumor size was measured with a sliding caliper every 5 day after injection. The tumor volume (V) were calculated using the following formula: V (mm^3) = V (mm^3) = π × width (mm) × width (mm) × length (mm)/6.
